# Understanding Independence: Board of Directors and CSR

**DOI:** 10.3389/fpsyg.2020.552152

**Published:** 2020-12-17

**Authors:** Reyes Calderón, Ricardo Piñero, Dulce M. Redín

**Affiliations:** ^1^Universidad de Navarra and Universidad Francisco de Vitoria, Madrid, Spain; ^2^Department of Business, University of Navarra, Pamplona, Spain; ^3^School of Economics and Business of the University of Navarra, Pamplona, Spain

**Keywords:** board independence, independent director, CSR, corporate governance, virtue ethics, practical wisdom, integrity

## Abstract

On August Business Roundtable (2019), the Business Roundtable redefined the purpose and social responsibility of the corporation. Yet, this statement must be followed by substantial changes in the business models of corporations for it to avoid becoming empty rhetoric. We believe that the figure of the independent director may be one of the catalysts needed for this change of paradigm for corporations. In spite of the positive correlation between Corporate Social Responsibility (CSR) and board independence, the development of the independence of boards during the last decade has not lead to the expected CSR results. Academics and regulators point to a weak definition and the non-standardized measurement of both independence and board independence (BI) as one possible explanation, and agree that a broader definition is needed. This paper aims to contribute to this debate. We develop a second-generation definition of independence based on a positive approximation to the concept by integrating an Aristotelian perspective of virtue ethics with the best practices of corporate governance. Thus, we define independence as a virtue guided by practical wisdom, that implies autonomy and autarky and which enables a person to act with integrity, fairness and truthfulness. In the context of corporate governance, independence is associated with an honest disposition to serve. Our proposal has political implications for supervisors that make decisions relating to the suitability of board members.

## Introduction

On August 19, Business Roundtable (2019), 181 top CEOs from the Business Roundtable (BRT) signed a statement that redefined the main purpose and social responsibility of the corporation. While a previous declaration in Business Roundtable (1997) fostered shareholder primacy, defending Friedman (1970)’s view that the only responsibility of business is to increase profits, the new guidelines seek to benefit all stakeholders, taking into consideration customers, employees, suppliers, community members and shareholders. Such corporate purpose is broader and more complete, closer to creating long-term value and shared prosperity in a sustainable way. This change of paradigm can be understood as a response to rising challenges to the social legitimacy of corporations in the wake of widespread inequality and environmental degradation.

Although the BRT statement is a promising start, it needs to be followed by substantial changes in the business model of many of the companies of the signatories. Otherwise the statement is just empty rhetoric (Bebchuk and Tallarita, 2020). Corporate governance cannot be alien to change and it must embrace instruments that make it more sensitive to its social and environmental impact.

Corporate Social Responsibility (CSR) initiatives are strategic by nature. As such, they fall under the mandate of the board of directors in a critical way (Gordon, 2007; Fassin and Gosselin, 2011). Indeed, theoretical and empirical literature have shown a positive correlation between CSR initiatives and the increased role played by independent directors (Chang et al., 2012). In a survey of 307 board members, Ibrahim et al. (2003) found that independent directors exhibit greater concern about the discretionary component of CSR. Webb’s (2004) analysis suggests that CSR is associated with independent and women directors. In other empirical study, Harjoto and Jo (2011) also discovered the CSR choice is positively correlated with Board Independence (hereafter BI), one of the essential characteristics of an effective board. Htay et al. (2012) found a similar correlation, this time with environmental aspects of CSR.

Independent directors are neither executive nor proprietary. Initially, within the framework that the purpose of the corporation was to create value for the shareholders, independent directors were meant to represent all those shareholders not represented on the board. However, in the context in which the company acquires more responsibilities, beside profits, expanding the field of action to all stakeholders on whom it has an impact, independent directors are entrusted with the responsibility to serve those stakeholders not represented in the board, taking into account their interests, needs and aspirations. Thus, when directors perform their duty well, the composition of the board of directors should improve CSR practices. This is attested by different studies (Haniffa and Cooke, 2005; Cai et al., 2006; Rouf, 2011) that, however, indicate that the progression is not as expected (Cf. Cuadrado et al., 2015).

Since the financial crisis in 2008, and thanks to general regulatory requirements and shareholder pressure, BI has become one of the most recommended practices in financial corporations (Pathan, 2009; Mehran et al., 2011; Sharpe, 2011; Adams and Mehran, 2012). Considered as an antidote to new corporate crises, independent members of the board have increased exponentially in financial corporations, reaching up to 70–85% in the United States (Adams, 2012) with similar figures in non-United States and some OECD countries (De Andres and Vallelado, 2008). In the context of the United States, Gordon (2007) shows that the presence of independent board members has augmented from 20% (in the 1950s) to 75% (in the 2000s). The increase is also exponential in non-financial large public corporations (Htay et al., 2012; Walls and Hoffman, 2012).

Given the positive correlation between BI and CSR, the growth of BI should be good news for social responsibility as it may be one of instruments with which to foster the balancing of stakeholder interests in current business models, as demanded by the BRT statement. However, the expected CSR results have not been produced. In the search for answers, (Bartukus et al., 2002; Cho and Kim, 2007; Arora and Dharwadkar, 2011; Chang et al., 2012), academics and regulators have pointed to a weak definition and measurement of both BI and independence as one possible explanation, and agree that a broader definition is needed (Joseph et al., 2014) especially if the social purpose in organizations needs to advance by integrating profits with other business purposes.

When it comes to BI, the literature focuses on its current definitional shortcomings. To start with, purely quantitative descriptions for it (understood as the ratio of outside/independent directors to inside directors) are criticized as inadequate. Adams (2012) emphasizes that this ratio alone is not a sufficient metric of “good governance”, and Rao and Tilt (2015) highlight the importance of using more qualitative methods. Similarly, Roberts et al. (2005) argue that having high numbers of independent members alone is of little use, as they must be active in order to be effective. Following these arguments, John et al. (2016) emphasize the importance of the “quality” of these independent directors, and recommend developing new and effective measures for it. Mehran et al. (2011), in turn, discuss whether board members can indeed exercise *intellectual independence* in the performance of their duties.

As for independence itself, the search for a global and consistent definition has been eluded both by regulators and academics (Hooghiemstra and van Manen, 2004). We have identified two main approaches currently used to consider someone to be independent: one based on the status and the other based on the context. The status-based approach – used by the Sarbanes-Oxley Act (SOX) or Nasdaq–, describes an *ex ante* materiality; the contextual approach – followed among others by the European Central Bank (ECB) (Rodrigues, 2008; Sharpe, 2011) – distinguishes between formal independence and Independence of Mind (IoM). Although with a different focus, both approaches depend solely on negative and passive descriptions to define the independence of members, whether as “outsider,” “non-executive,” “non-employee,” “non-interested,” or “disinterested.”

We believe these first-generation definitions are incomplete and they fall far short of enabling the change in corporate governance decisions that the BRT requires. A more complete definition should aim for an integral approach of the individual, thus taking into account the past, the context, and the objective and subjective qualification of the person (Kaptein and Wempe, 2002; Calderón et al., 2018), and make independent directors truly empowered to make decisions with a strong ethical, as well as economic, consequences.

Evidence has proved that whenever executives failed, it was rarely because of a lack of expertise or technical training, but due to interpersonal skills and practical wisdom (Bennis and O’Toole, 2005). In this line, we propose a second-generation definition of independence that hinges on its positive and active role, integrating an Aristotelian perspective of virtue ethics with the best practices of corporate governance. We define independence as a virtue guided by practical wisdom, that implies autonomy and autarky and which enables a person to act with integrity, fairness and truthfulness. In the context of corporate governance, independence is associated with an honest disposition to serve.

Practical wisdom (*phronesis)* is the habitual disposition of doing the right things the right way (Shotter and Tsoukas, 2014). Judgment is the inherent action of independent directors and *phronesis* the virtue of “good judgment” (Solomon, 1992: 328–29). Practical wisdom develops the moral character, the priority lies with whom directors become as a result of their actions. Independence implies a way of being that is intimately related to the concepts of autarky and autonomy. This is an acquired, conquered and configured capacity aided by both wisdom and will, which translates into three fundamental characteristics that are exhibited by the person that seeks to be independent: integrity (Tanner et al., 2019), fairness (Diao et al., 2019; Trinh, 2019) and truthfulness (Zagzebski, 1996). In addition, the independent person that occupies an executive responsibility also exhibits an honest disposition to serve (Heintz et al., 2016), since governance is synonymous with service and thus requires continuous learning, training, updating and commitment. Our contribution becomes especially important to guide supervisors – such as the ECB – that make decisions relating to the suitability of board members, with independence being one of its key elements. Yet, it is not our intention to provide a step-by-step guide to evaluate the degree of independence of a candidate but to set the general guidelines that should inspire normative efforts to achieve this.

This paper proceeds as follow. Section “The Current Situation of Independence: The First Generation” offers a critical description of the first generation of independence and its two main approaches. Section “The Second Generation: A Positive Approach to Independence” analyses the key dimensions of independence and it proposes a positive definition of the concept, referred to here as the second generation of independence. Section “Conclusion” summarizes the main conclusions of the paper.

## The Current Situation of Independence: The First Generation

BI has exponentially increased in financial and large public corporations, without a debate about its defining conditions. As Fairfax (2010:133) details “the term *independent director* has no uniform definition; instead judges and legislators define the term differently. Moreover, the term is used differently in various contexts.”

Belonging to a board of directors, whatever the category of the director, implies that person is bound to a duty of care and a duty of loyalty, the purpose of which is to align the interests of the administrators and the shareholders with those of the stakeholders, with the objective of creating value and ensuring its distribution, as well as giving confidence to the market. To carry out these duties, the members of the board, whatever their category, must perform their functions and hold their opinions independently, that is, without being conditioned by the interests of others and with the strength or soundness of character that their position requires.

However, in corporate governance when we talk about an independent director, we do not refer to that generic independence but to a more specific and regulatory one. Accordingly, two different approaches have been adopted to describe an independent member: the status-based approach and the contextual approach which together formed the first generation of independence.

### The Status-Based Approach to Independence

In response to the Enron and subsequent debacles, and to the recurrent implication of executive directors in the scandals, regulation adopted an incisive spirit of “command-and-control” with the aim of mitigating agency costs and restoring lost integrity (Thibodeau and Freier, 2014). In this context, it was understood that the most outstanding characteristic of an independent member to inspire confidence as a controller was precisely to be able to ensure that she had no relationships or potential conflicts of interest with the firms she was trying to supervise. In this line, SOX, which was one of the major regulatory responses in this spirit, described the independent director through the status approach.

Most regulators from the Anglo-Saxon tradition follow the line marked by SOX, such as NASDAQ or the New York Stock Exchange (NYSE), and understand that the distinguishing characteristic of the independent director is the status of outsider, analyzed from the point of view of her past relationships with the company. Thus, the status-based approach defines independence as a sign of non-dependence referring to the independent director as an “outsider,” “non-executive,” “non-employee,” or “disinterested” board member. The non-dependence status, which can be certified *ex ante* by looking into the past and with no reference to the context of the transaction, reflects the absence of both financial links with the corporation and family ties with management, and it must be ratified by the board.

The NYSE and NASDAQ stipulate that an independent member cannot have any “material relationship” with the listed company [NYSE, §303A (2) (a)], meaning not only that she should not be directly related as a partner, shareholder or officer, but also that she should pass an exhaustive test of materiality. The definition of materiality, assessed at the time of entry of the new member to the board, requires verifying (a) the present and (b) past relationships of the individual, and (c) of her family members or their associates to the corporation. Regarding past relationships, materiality is described both by the type of relationship itself, by the intensity or by the years elapsed since those relationships ceased to the present. Each regulator develops its own materiality test along these lines.

For instance, the NASDAQ materiality test considers that any trace of previous service to the corporation will be eliminated after 3 years instead of five; it uses a broader definition of family member, which includes any person who is related by blood, marriage or adoption or who has the same residence; it recognizes dependency in any person who has received more than $60,000 from the company for consulting or other services in the previous 3 years, or when the director had been a partner, officer or majority shareholder of a company that made or received payments above a certain amount in the previous 3 years.

This view of non-dependence is directly correlated with the agency problem, perhaps the central problem of corporate governance in banks (Rodrigues, 2008), which has been found at the core of many scandals (Bainbridge, 2002).

Large public corporations and banks are characterized by fragmented and dispersed ownership and the lack of power, incentive or means to monitor the actions and decisions of the managers (Mehran et al., 2011). In this context, managers may act in their own best interest rather than in the interest of the firm (Gilson and Schwartz, 2015). Independent members should contribute (a) to align the financial interests of executives with those of shareholders, decreasing agency costs and “opportunistic” behavior and (b) to preserve the long-term interest of the company when conflict between majority and minority shareholders arises, curbing the extraction of private benefits to the detriment of other stakeholders, and (c) to align social responsibility (Courteau et al., 2017).

Independent members should help shareholders to solve this agency problem because they have no “need or inclination to stay in the good graces of management and (…) will be able to speak out, inside and outside the boardroom, in the face of management’s misdeeds in order to protect the interests of the shareholders” (Clarke, 2007: 84). As Dallas (2003) emphasizes, while insider-dominated boards perform the role of formulating corporate strategy more effectively, an outsider-dominated board is preferred to perform the function of management monitoring more effectively.

Judged from a historical perspective, the status-based approach contributed considerably to the good corporate governance of the entities enhancing their materiality tests in order to overcome the ambiguous nature of these subjects. However, this approach has been criticized accordingly for its negative perspective and especially for its static and *a priori* assessment.

Indeed, in the context of the resolution of agency problems, independence, that is non-dependence, is understood as distance and even as preventive mistrust. However, necessarily over time, an independent director will acquire familiarity with the company and its executives, which would prevent her from being qualified literally as an outsider, or as a disinterested member. This familiarity, increasing over time, nevertheless has a positive interpretation, since with a closer knowledge of the company and its management, a constructive director may contribute to a greater degree and with higher quality in the decision-making process. In sum, the fact that a person fulfils some criteria *ex ante* does not guarantee an *ex post* independent behavior. As Sharpe (2011) highlights, the independence that is only verified *ex ante* cannot be more than cosmetic. As Roberts et al. (2005, pp. 16–17) point out, under certain circumstances, independence will not be “an independence *from* executives, but rather exercising an IoM *in support of* executive and company success. In bad times, non-executives can support executives with suggestions and advice about how to weather a storm. In good times, they can act as a guardian against executive exuberance.”

Furthermore, taking into consideration the increasing complexity of the corporate activity and its opacity, a complete lack of familiarity with the subject would be counterproductive but a strong familiarity with it would result in the rejection of many candidates (Adams, 2012).

On the other hand, we can argue that non-dependence does not guarantee a correct decision if it is not accompanied by other factors. In other words, being non-dependent is not always or even in any case, synonymous with being independent (Fairfax, 2010). In that vein, Sharpe (2011) distinguishes between a *cosmetic independence* that takes into account only a corporate relationship with the corporation and not the tools a director needs to achieve substantive independence, and a high-performance independence, which includes the critical components of good decision-making: time, information and knowledge.

The argument that materiality must be completed with other elements has opened the door to a more contextual view of independence. We agree that the status-based approach falls short since it does not guarantee that an independent director would act with the required independence of character. However, we believe that the materiality of independence, defined unambiguously in negative terms, becomes a prudent safeguard of the preliminary conditions. That is, materiality is a necessary condition, but it is not enough and, therefore, it is partial and incomplete (Bainbridge, 2002). In the negative terms and as a starting condition, independence points to *someone who is shown as independent*, who *seems to be* independent because certain circumstances occur around her, but albeit necessary, this approximation falls short.

### The Contextual Approach to Independence

Considering an *ex ante* definition of independence as essentially impossible, the contextual approach proposes the analysis of each conflict of interest, observing a combination of factors as an alternative to the rigid and pre-programmed status view. Hence, it distinguishes between an independent and an interested member. A director is interested in a given transaction if she stands to gain monetarily from it in a way that other shareholders do not or if she expects to derive any personal financial benefit from it in the sense of self-dealing (Clarke, 2007). The focus is not reduced to the area of financial interest. It must be examined whether a director is in some way committed to, or exhibits affinity toward another individual who is interested, or whether she is influenced by personal considerations. Therefore, circumstances such as belonging to the same university, being involved in the same charitable donations, sharing a common religion or friendships may be factors to be taken into consideration.

The approach endeavors to ensure the “no interest” of the director, but not her disinterest. If independent directors must address complex instruments and trading activities (Andrés et al., 2012) they must be professionals in an area which is akin to the financial sector, therefore a rigid catalog of *ex ante* materiality cannot contemplate all the situations that are likely to take place *ex post* (Langevoort, 2001).

It is this line of thought which underpins the doctrine of supervisors such as the ECB, responsible for the prudential supervision of credit institutions located in the euro area and participating non-euro area member states. The ECB articulates the Single Supervisory Mechanism (SSM) with internal governance being one of the top supervisory priorities. The ECB must adopt decisions relating to the suitability of board members, and independence is one of the elements which it has to watch over.

Starting with a regulatory position in line with the status-based approach, in recent years, the ECB evolved its stance developing a new approximation around the concept of IoM. It tries to overcome the problems generated by the approach to independence based exclusively on the status of the director.

The (European Central Bank [ECB] (2016): 8) first introduced this contextual approach of independence in its publications in 2016 highlighting that “from a theoretical point of view, independence should be understood both as ‘formal’ independence and ‘in mind’.” “Formal” independence was associated with the proportion of independent directors on the board, whereas independence “in mind” is identified with the absence of potential conflict of interests. Initially, no mention is made to whether that condition must hold *ex ante* or *ex post.*

One year later, the SSM retrieves and expands the concept of independence. It is interesting to highlight that the SSM makes a small twist in the terminology that it uses, and it starts to speak of independence “of mind” rather than the independence “in mind”. This tiny detail has major philosophical implications since the former alludes to the behavior or the action of the individual, and the latter to her character. The SSM defines “to act with IoM” as “being able to make sound, objective and independent decisions” (European Central Bank [ECB], 2017a, p. 15) and it points out that IoM can be affected by conflict of interests either actual, potential– i.e., reasonably foreseeable–, or perceived–i.e., by the public.

In this context, the supervised entities are required to adequately address any sort of conflict of interest and explain to the competent authority how it is being prevented, mitigated or managed. Interestingly, the SSM emphasizes that having a conflict of interest does not necessarily imply that the person cannot be considered suitable as an independent director as long as the conflict does not impose a material risk and it is adequately prevented, mitigated or managed according to the written policies of the supervised entity. The SSM states that any material conflict of interest should be assessed on a case-by-case basis. Hence, it leaves a door open that invites one to look beyond the status of the director.

Moreover, for the first time, a temporal view is introduced. The SSM (European Central Bank [ECB], 2017a) highlights, in a non-exhaustive manner four potential sources of conflicts of interests – with respect to the corporation, including the parent or subsidiaries – that are evaluated within different time frames. Personal and financial causes are evaluated in the present, whereas professional or political sources of conflicts are binding within a 2-year lag. Furthermore, these sources apply not only to the independent directors themselves, but also to the circle of their close personal relations.

In a later report (European Central Bank [ECB], 2017b), the SSM goes one step further and explicitly differentiates between the notion of IoM and the principle of “being independent;” the former is applicable to all members of the management body of an institution whereas the latter is required for certain managers in their supervisory function. Hence, the fact that a member is considered as “being independent” does not mean that she should automatically be deemed to be IoM as the member might lack the required behavioral skills. The SSM (European Central Bank [ECB], 2017b) clarifies that acting with IoM is actually a pattern of behavior, revealed particularly during discussion and decision-making, which enables the making of sound, objective and independent decisions and judgments. “Being independent” means that the person does not have any present or past relationship or connection of any nature with the institution that could influence the capacity of the member to act with IoM.

In that same report (European Central Bank [ECB], 2017b), the SSM provides some criteria for assessing the IoM: it must be evaluated according to two main conditions; behavioral skills and the absence of conflicts of interests, which would handicap the ability to act with genuine independence. At this point it can be noticed how the SSM expands the context of independence not only outward–toward the circumstances surrounding the director–, but also inward, appealing to the virtues and the traits of the character of the individual. In particular, it points out that in order to have IoM a director should exhibit courage, conviction and strength, having the capacity to ask questions and demonstrate resistance to “groupthink.”

When it comes to independent directors, the SSM (European Central Bank [ECB], 2017b) recalls that they should ensure that the interests of stakeholders are taken into account in the discussions and decision-making. The formal independence of the directors is defined in negative terms, again by way of their status, so that if the person “is not” anything that is specified by SSM, she is deemed to be independent.

The mere fact of meeting in a positive sense one of these requirements does not automatically qualify a person as not being independent; in that case, the institution may demonstrate to the competent authority the reasoning why the person is able to act with IoM. Hence, the SSM affords vital importance to the context and the evaluation of the particular circumstances in which the director makes decision and acts beyond their status.

In one publication concerning good governance practices, the SSM (European Central Bank [ECB], 2018) recalls the importance of a contextual approach: “Banks need to have a sufficient number of independent members on their boards. These members play a key role in providing the necessary checks and balances. However, formal independence is not enough in itself: all board members need to be independent thinkers too. In board discussions, the view of each board member must count. This is a prerequisite not only for sound collective decision-making, but also for fostering critical thinking and diversity, which are essential qualities in counterbalancing the risk of groupthink.”

Beyond reconciling the status and the context, the great contribution of the SSM approach is to direct the debate toward the essence of the individual who performs the role of the independent director. The are two landmarks in this process. First, when the SSM–albeit rather timidly and perhaps unwittingly – raises the question of whether the directors should act with independence “of mind” or “in mind,” and second, when it expands inward, to the interior of the human being, the context in which independence ought to be evaluated, pointing out some virtues and character traits that are deemed desirable. This point directly opens up a gap for a third way which should unite the condition of the character and the performance of the individual, which must encompass both the status and the context.

## The Second Generation: A Positive Approach to Independence

The first generation of independence is characterized by a person *not* immersed in a conflict of interests, someone who does *not* depend on another or who does *not* undergo interventions by another. In sum, the independent member is defined as the one that is *not* subject to another.

A definition of independence based on the status that only takes into account the potentially harmful relationships between the director and the corporation that may have taken place prior to her entry onto a board is completely biased, describing only a mere appearance of independence (Cf. Kaptein, 2018) and it does not guarantee that the director will act with independence. While the concept of status is valid as a condition, as a first step or prudent safeguard against agency problems, it is not valid to build a complete definition of independence around it (Bainbridge, 2002).

Advancing a further step, by adding *a priori* conditions which would seem to guarantee that the director is not disinterested – in spite of not having a direct interest–, allows the contextualization of the condition of independence and ensures that the decision-making process is more effective (Rodrigues, 2008; Fairfax, 2010). However, the sort of independence in decision making that should be incarnated by the independent director is not guaranteed by this approach either.

We believe that a complete definition of independence should be based on an integral vision of the person, encompassing her virtues and character (Cf Kaptein and Wempe, 2002) beside her formal qualifications. Neither things nor words are “independent.” Only people can be and also not be. Avoiding certain positions–status–, or meeting certain conditions–context–, enables the set of potential candidates to be ranked, which is a filter that shareholders must demand to shield their interest in dealing with agency problems, but it does not imply *being* independent as independence is an eminently practical condition that manifests itself in a “way of acting,” in a “way of being in the world,” and finally it reflects a “way of being.” Hence, the second generation of independence must be described in positive terms. We defined independence as a virtue guided by practical wisdom, that implies autonomy and autarky and which enables a person to act with integrity, fairness and truthfulness. In the context of corporate governance, independence is associated with an honest disposition to serve.

Independence demands practical wisdom (*phronesis).* Aristotle defines *phronesis* in the Nicomachean Ethics (henceforth NE: 1144a) as the virtue of choosing the suitable means to the right end (Aristotle, 1991). But what are the distinctive features of practical wisdom that make us associate it with the ideal of independence in corporate governance? We follow through Sison and Hühn (2018) to highlight the main characteristic of practical wisdom for corporate governance. First, practical wisdom is a moral virtue that implies doing the right thing, the right way, for the right purpose, and in the right circumstances (NE: 1126b). That is, performing the morally right action correctly. It establishes habitual alignment between proper perception, rational deliberation, choice and behavior (NE: 1140a–b). Second, practical wisdom expresses normativity beyond moral absolutes (NE 1110a) and general rules (NE 1137b). It implies accepting rules or codes, but only as general guidelines, since they are never sufficient. Proper rule-following invariably needs “prudential judgment” in its interpretation and implementation since rules are not perfect, and they may not foresee all the contingent circumstances. Practical wisdom studies the norms applicable to a situation, the circumstances or features of a problem, and decides how to proceed (Moberg, 2007). Third, practical wisdom exercises an integrative function between dispositions to action and virtues (NE 1145a); without it, no genuine virtue exists. Fourth, practical wisdom constitutes a perceptual subjective standard of who the agent has become as a result of previous action rather than an objective, external rule of behavior available to any independent observer. Finally, Aristotle lists practical wisdom among the character traits (*ethos*) that a speaker must have to convince or persuade an audience (Rhetoric: 1356a). Together with virtue (*arête*) and goodwill (*eunoia*), practical wisdom allows speakers and leaders to form correct opinions over concrete, contingent issues, enabling them to express views justly and fairly, and ensuring sound advice to listeners. They form the basis of a person’s credibility and trustworthiness.

Independence must be synonymous with freedom, self-government, self-control (Cf. Rua et al., 2017). An independent person is thus the one who has the possibility to present and sustain her opinions without interference or intervention from others. This is well expressed by Langevoort (2001) when he points out that independence should not consist only in the lack of financial ties with the administration, it also includes a clear expression of the will to provide a high degree of rigor and skeptical objectivity regarding the evaluation of the administration of the company, and its plans and proposals. Both independence and autonomy are acquired, conquered capacities, configured in the full deployment of wisdom and will. It is a deployment that, by assuring that all the necessary elements are possessed to carry out the action to the end, is carried out firmly, in the sense of solidity, and morally right with the help of practical wisdom. Practical wisdom guarantees a proper alignment between this sort of IoM and the action of the independent director. This task also requires autarky and autonomy which could be understood as an intermediate step toward independence.

From a theoretical perspective, autonomy reminds us of the capacity to empower ourselves with laws of our own, that is, that the person freely and voluntarily imposes upon herself a criterion of action, a norm that guides her behavior. The key to autonomy is in the action of giving to oneself, not so much in the exercise or in the compliance of that norm that we have given ourselves. The strength of that action rests in the fact that the individual considers that this norm has a foundation with which she agrees. The importance of the foundation also lies in its scope: the basis can be subjective, objective, intersubjective. As an objective principle, the norm is self-imposed upon the will; as a subjective principle, the norm is an exercise of one’s will; as intersubjective, the key lies in the conviction that it will be assumed by a good number of people who will appreciate in the standard not just a mere particular occurrence, but a principle that can be assumed by a community as a whole. In this sense, independence is close to being a categorical imperative in which what governs the action is not a particular interest, even if it is legitimate, but in which the independent action is an end in itself.

The practical consequence of autonomy is autarky in a three-fold sense. First, because autonomy puts us in a position to exercise self-government. Second, autonomy also allows the subject of the action, by virtue of the assumption of her own criterion, to exercise a detachment from the outside. Independence is like an inner conquest that does not renounce to the exteriority, to the other, but, having examined it all, it exercises the decision to separate oneself from that exteriority. And, third, the most practical consequence is the exercise of action itself: not only the examination of the conditions of the action, but the exercise itself, its implementation, taking as a horizon that the virtue of governance is, in reality, the governance of virtue (Moore, 2012).

Independence leads to three fundamental characteristics that chatacterize the actions of the independent director: integrity, fairness and truthfulness.

Integrity is a kind of completeness that results from adequate intellectual and technical formation and, at the same time, denotes a moral significance, in the sense that whoever is integral can never be someone outside of an ethical code and, therefore, whose action always has consequences that affect other people. Integrity derives from the social nature of independence.

Fairness is a consequence of the proportionality and balance that reign in the decision making and in the execution of the acts of the independent agent. Independent is never synonymous with “fickle” and much less “arbitrary” (Diao et al., 2019; Trinh, 2019). A person who acts independently, is someone who weighs the pros and cons; who examines carefully and in a balanced way the factors that affect a certain issue; who deliberates by showing prudence, without resorting to hasty actions that can lead to or facilitate error; who with respect – and even cordiality – is able to put themselves in the shoes of the other, with empathy and delicacy.

In addition, independence is necessarily linked to truthfulness, since only a precise knowledge of reality enables the formation of a solid criterion of action. Of course, our rationality is limited and our knowledge may not be optimal, but what is undeniable is that we can do no less than endeavor to acquire the maximum possible correct, rigorous, contrasted information.

A final nuance associated with the independent person who reaches executive responsibility levels is the disposition to the service. Governance implies service and that task requires continuous learning, training, updating and commitment.

In this positive perspective, independence is simultaneously a value and a virtue. It is a virtue insofar as it is a human excellence and it produces positive feedback among other virtues found in multiple operational trajectories. It is not possible to be independent without being practically wise (*phronimos*), just, temperate and courageous at the same time. Hence, it is not possible to be a good independent director without trying to render different stakeholders their due, being moderate in the pursuit and use of resources, and persevering in the struggle against obstacles. Independence as a virtue leads to flourishing (NE: 1140a), the final end of human beings which is essentially social and relational, not individualistic and utilitarian. From an Aristotelian perspective, the goal of the independent director should refer to how to make people capable of joint performance so that the firm, as an economic unit, can contribute to flourishing (*eudaimonia*) in political communities, the ultimate end of human existence. The purpose of the firm displays the characteristics of a common good: something that can only be accomplished if all group members do so simultaneously (Sison and Fontrodona, 2012), which undoubtedly resonates to the principles of the BRT statement.

Independence is a virtue and as such it is voluntary (NE: 1114b). It is not a simple act of doing, but it is exercised because of the goodness of the action itself, and in this sense, it is the best clarification of an action not mediated by any conflict of interest – which is the premise for formal independence. The action of the independent person is not motivated by secondary interests, but rather it is driven by the final end (*eudaimonia*). Independence should overcome the tyranny or pressure exerted by other interests which are not ordered means to lead the corporation toward the common good of all its members.

Independence becomes a value in itself because it turns the person who possesses it into someone “special,” who is supposed to have a strong moral character as well as aptitude, skill, efficiency, and display exemplarity and leadership (Neubert et al., 2009). Value and virtue can only be understood if they are incarnated in the practical life of a specific individual, Therefore, there is no gap between being independent and acting with independence (Sen, 2009: 158), because we can only identify the genuine independent person by her actions; without action, there is no independence because there is no practice. Thus, independence as a way of being – and not only as a way of acting – is intimately related to integrity.

The second generation of independence is radically linked to an ethical and psychological perspective. The “*ethos*” is not only a certain type of character but above all, a way of being that directs the way of acting (Chun, 2005). Perhaps it is to this dimension that the European Central Bank [ECB] (2016) refers when it mentions the aspiration to achieve an “independence *in* mind” that goes beyond mere formality, which finds its meaning more than in normative codes, but in concrete behaviors of specific people.

For the second generation, we have defined independence as a virtue guided by practical wisdom, that implies autonomy and autarky and which enables a person to act with integrity, fairness and truthfulness. In the context of corporate governance, independence is associated with an honest disposition to service. In the end, independence is an ideal that is only incarnated in people, and these are the clearest expressions of fragility. There is no independence without independent directors and these are neither automatons nor infallible, but they have the conditions and the position to lead the change in business models that the BRT statement demands.

## Conclusion

Corporate Social Responsibility has been correlated with BI and, in recent decades, the value and weight of BI has become imperative in corporate governance. However, CSR has not developed at the same pace. One explanation is the lack of a consistent definition that identifies the characteristics of independent directors. Traditionally, there have been two different approaches to identify a director as being independent: the status-based approach – used by SOX and Nasdaq–, which describes an *ex ante* materiality, and the contextual approach – followed by the ECB – which distinguishes between formal independence and IoM. Both approaches are part of a first generation of definitions that are characterized by a negative conception of independence, centered on its supervisory function, that turns out to be partial. They do not guarantee that a director that qualifies as independent actually behaves with the independence that her position demands. We believe that a complete definition of independence should be based on an integral vision of the person, encompassing her virtues and character, besides her formal status and the contextual circumstances, because there is no independence without independent people. This paper provides a second-generation definition of independence based on a positive approximation to the concept. We defined independence as a virtue guided by practical wisdom, that implies autonomy and autarky and which enables a person to act with integrity, fairness and truthfulness. In the context of corporate governance, independence is associated with an honest disposition to serve.

We believe that if corporations are able to sustain their BI with independent members inspired by this second generation of independence, they will be able to implement effective changes in their business models to align their corporate and social purpose with the demands of the BRT statement.

Following the Aristotelian social tradition, corporate governance refers to how the corporation, as an economic unit, could contribute to flourishing (*eudaimonia*) in political communities, the ultimate end of human existence. The purpose of the firm displays the characteristics of a common good: something that can only be accomplished if all group members do so simultaneously (Sison and Fontrodona, 2012). We believe that this account of the purpose of the corporation is in line with the principles set by the BRT statement, and it stresses the political dimension of business. Business should not be conceived independently of society, and thus corporate directors must display an honest disposition to serve to all the stakeholders on whom the corporate activity impacts.

Independence requires practical wisdom. *Phronesis* guarantees that independent directors look for goods of effectiveness – i.e., external standards of success such as profits, market share, share price – in so far as they enable goods of excellence – i.e., the virtues people develop in support of the firm which are conducive to flourishing (Sison and Hühn, 2018). Hence, it debunks Friedman (1970)’s thesis that stresses the supremacy of shareholders’ interests.

Understanding independence as a virtue implies that it is voluntary, a good in itself, perfective of the individual that acts with independence. The actions of the independent director are not mediated by any conflict of interest – the goal of formal independence–, but rather they are motivated by the final end (*eudaimonia*). Accordingly, the second-generation of independence will be more effective than its first-generation counterpart as an antidote to corporate scandals, which has been the main concern of the regulatory efforts that developed the figure of the independent director.

Finally, independence is associated with a set of virtues that reinforce each other in a positive feedback effect. An agent may not be independent without being prudent, courageous or a person of integrity and justice. Hence, a good independent director will strive to render the various stakeholders their due, being moderate in the pursuit and use of resources, and look for the wellbeing of the communities linked to the corporate activity and society as a whole.

Future research should focus on designing specific selection criteria and evaluation methods for second-generation independent directors, regulatory reforms and policies to translate this second generation of independence onto boards, and strategies to promote the development of a corporate culture that fosters a second generation of independence.

## Author Contributions

RC is professor of corporate governance and an independent member of some boards of directors. RP is professor of ethics and aesthetics. The research of DMR focuses on virtue ethics in business and management. All authors contributed to the article and approved the submitted version.

## Conflict of Interest

The authors declare that the research was conducted in the absence of any commercial or financial relationships that could be construed as a potential conflict of interest.
